# Deleterious *ZNRF3* germline variants cause neurodevelopmental disorders with mirror brain phenotypes via domain-specific effects on Wnt/β-catenin signaling

**DOI:** 10.1016/j.ajhg.2024.07.016

**Published:** 2024-08-20

**Authors:** Paranchai Boonsawat, Reza Asadollahi, Dunja Niedrist, Katharina Steindl, Anaïs Begemann, Pascal Joset, Elizabeth J. Bhoj, Dong Li, Elaine Zackai, Annalisa Vetro, Carmen Barba, Renzo Guerrini, Sandra Whalen, Boris Keren, Amjad Khan, Duan Jing, María Palomares Bralo, Emi Rikeros Orozco, Qin Hao, Britta Schlott Kristiansen, Bixia Zheng, Deirdre Donnelly, Virginia Clowes, Markus Zweier, Michael Papik, Gabriele Siegel, Valeria Sabatino, Martina Mocera, Anselm H.C. Horn, Heinrich Sticht, Anita Rauch

**Affiliations:** 1 Institute of Medical Genetics, University of Zurich, Zurich, Switzerland; 2 Faculty of Engineering and Science, University of Greenwich London, Medway Campus, Chatham Maritime ME4 4TB, UK; 3 Medical Genetics, University Hospital Basel, Basel, Switzerland; 4 Center for Applied Genomics, Children’s Hospital of Philadelphia, Philadelphia, PA, USA; 5 Division of Human Genetics, Children’s Hospital of Philadelphia, Philadelphia, PA, USA; 6 Neuroscience Department, Meyer Children’s Hospital IRCCS, Florence, Italy; 7 University of Florence, Florence, Italy; 8 Unité Fonctionnelle de Génétique Odellin, Hôpital Armand Trousseau, Paris, France; 9 Département de Génétique, Hôpital de la Pitié-Salpêtrière, Paris, France; 10 Faculty of Science, Department of Biological Science (Zoology), University of Lakki Marwat, Khyber Pakhtunkhwa 28420, Pakistan; 11 Shenzhen Children’s Hospital, Shenzhen, Guangdong, China; 12 Instituto de Genética Médica y Molecular (INGEMM), Unidad de Trastornos Del Neurodesarrollo, Hospital Universitario La Paz, Madrid, Spain; 13 Department of Clinical Genetics, Odense University Hospital, Odense, Denmark; 14 Nanjing Key Laboratory of Pediatrics Children’s Hospital of Nanjing Medical University, Nanjing, China; 15 Northern Ireland Regional Genetics Centre, Belfast Health & Social Care Trust, Belfast, Northern Ireland; 16 Thames Regional Genetics Service, North West University Healthcare NHS Trust, London, UK; 17 Institute of Biochemistry, Friedrich-Alexander-Universität Erlangen-Nürnberg (FAU), Erlangen, Germany; 18 Pediatric University Hospital Zurich, Zurich, Switzerland

**Keywords:** ZNRF3, microcephaly, macrocephaly, mirror phenotype, Wnt signaling, tumor suppressor gene, haploinsufficiency, dominant negative, adrenal insufficiency, congenital heart defects

## Abstract

Zinc and RING finger 3 (ZNRF3) is a negative-feedback regulator of Wnt/β-catenin signaling, which plays an important role in human brain development. Although somatically frequently mutated in cancer, germline variants in *ZNRF3* have not been established as causative for neurodevelopmental disorders (NDDs). We identified 12 individuals with *ZNRF3* variants and various phenotypes via GeneMatcher/Decipher and evaluated genotype-phenotype correlation. We performed structural modeling and representative deleterious and control variants were assessed using *in vitro* transcriptional reporter assays with and without Wnt-ligand Wnt3a and/or Wnt-potentiator R-spondin (RSPO). Eight individuals harbored *de novo* missense variants and presented with NDD. We found missense variants associated with macrocephalic NDD to cluster in the RING ligase domain. Structural modeling predicted disruption of the ubiquitin ligase function likely compromising Wnt receptor turnover. Accordingly, the functional assays showed enhanced Wnt/β-catenin signaling for these variants in a dominant negative manner. Contrarily, an individual with microcephalic NDD harbored a missense variant in the RSPO-binding domain predicted to disrupt binding affinity to RSPO and showed attenuated Wnt/β-catenin signaling in the same assays. Additionally, four individuals harbored *de novo* truncating or *de novo* or inherited large in-frame deletion variants with non-NDD phenotypes, including heart, adrenal, or nephrotic problems. In contrast to NDD-associated missense variants, the effects on Wnt/β-catenin signaling were comparable between the truncating variant and the empty vector and between benign variants and the wild type. In summary, we provide evidence for mirror brain size phenotypes caused by distinct pathomechanisms in Wnt/β-catenin signaling through protein domain-specific deleterious *ZNRF3* germline missense variants.

## Graphical abstract

**Figure:**
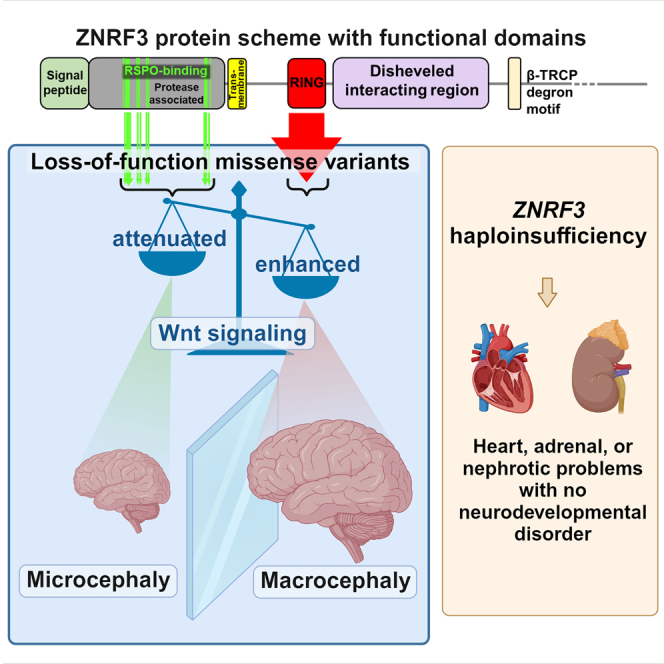

## Teaser

Boonsawat et al. establish germline missense variants in the tumor suppressor gene *ZNRF3* as a cause of neurodevelopmental disorders (NDDs) with microcephaly or macrocephaly, depending on the functional/domain-specific effects of the variants on Wnt/β-catenin signaling. This study finds that *ZNRF3* haploinsufficiency does not cause NDDs but rather other phenotypes.

## Introduction

Wnt/β-catenin signaling plays an important role in human brain development.^1^ Genetic variants affecting this signaling can cause either an abnormally small or large brain,^2^ both of which have been rarely shown to be caused by a single gene/region.^3^^,^^4^ Indeed, one study of *WDFY3* (MIM: 617485) germline variants has linked upregulation of the Wnt/β-catenin signaling to microcephaly and downregulation to macrocephaly with unclear molecular mechanism.^3^

Here, we demonstrate such opposing effects on human brain size by distinct missense variants in the gene zinc RING finger 3 (*ZNRF3* [MIM: 612062]) and show that an increase in Wnt/β-catenin signaling, in contrast to the aforementioned study,^3^ leads to macrocephaly, while a decrease in Wnt/β-catenin signaling leads to microcephaly. *ZNRF3* encodes a transmembrane E3 ubiquitin ligase containing several domains, including, among others, an R-spondin (RSPO) binding domain and a RING-type E3 ligase (RING). *ZNRF3* has been shown to be expressed upon Wnt activation, forming a dimer at the plasma membrane and targeting the Wnt receptor Frizzled for ubiquitination and degradation thereby functioning as a negative-feedback regulator of the Wnt signaling.^5^ ZNRF3-mediated inhibition of the Wnt/β-catenin signaling can be counteracted by RSPO, a secreted protein that binds to ZNRF3 and induces its membrane clearance.^5^

Wnt/β-catenin signaling plays a crucial role in embryonic development and adult tissue homeostasis, but constitutive activation of Wnt/β-catenin signaling can lead to a variety of cancers.^6^^,^^7^ Accordingly, prolonged activation of Wnt/β-catenin signaling due to somatic alterations of *ZNRF3* has been linked to adrenocortical carcinomas in human.^8^ Moreover, ZNRF3 downregulation has also been observed in many other human cancer cell lines or tissues.^9^^,^^10^^,^^11^^,^^12^ In osteosarcoma cells, ectopic expression or knockdown of *ZNRF3* resulted in compromised or enhanced cell proliferation, respectively.^9^ In mice, complete knockout of *Znrf3* led to perinatal death,^5^ while heterozygous mice survived but developed a range of gonadal abnormalities.^13^

To date, no clear disease association exists for *ZNRF3* germline variants. Here, we describe 12 individuals with germline missense, truncating, or large in-frame deletion *ZNRF3* variants and assessed genotype-phenotype correlation. While the four individuals with *de novo* truncating or *de novo* or inherited large in-frame deletion variants showed a variety of non-neurodevelopmental disorder (NDD) phenotypes with uncertain pathogenicity, the eight individuals with *de novo* deleterious missense variants had NDDs with distinct brain phenotypes and distinct abnormalities in our functional assays. We show that a variant from a microcephaly-affected individual as well as a designed variant disrupted the RSPO binding site and decreased Wnt/β-catenin signaling, while variants from macrocephaly-affected individuals perturbed the RING domain and increased Wnt/β-catenin signaling in a dominant negative manner. Our findings suggest opposite mechanisms via Wnt/β-catenin signaling by which variants in a single gene exert its effect to mirror brain phenotypes in a protein domain-dependent fashion.

## Subjects, material, and methods

### Affected individuals and exome sequencing

Affected individual 1 (I-1) was part of an exome sequencing study to unravel the genetics of microcephaly (Table 1) with ethical approval by the ethical commission of the canton of Zurich.^14^ I-3 and I-8 were part of the Deciphering Developmental Disorders study.^15^ I-2, I-4, I-5 to I-7, and I-9 to I-12 were recruited via GeneMatcher database.^16^ Written informed consent for publication of clinical information and/or photographs were obtained.

**Table 1:** Summary of *ZNRF3* variants and clinical findings

**Affected individual**	**I-1**	**I-2**	**I-3**	**I-4**	**I-5**	**I-6**	**I-7**	**I-8**	**I-9**	**I-10 and I-11 (father, son)**	**I-12**
GRCh37(chr22) NM_001206998.2 NP_001193927.1	c.311T>C (p.Leu104Pro)	c.536C>T (p.Pro179Leu)	c.878G>A (p.Cys293Tyr)	c.887G>A (p.Cys296Tyr)	c.920G>A (p.Arg307Gln)	c.956G>A (p.Cys319Tyr)	c.965C>G (p.Pro322Arg)	c.1000C>T (p.Arg334Trp)	c.190del (p.Val64Cysfs^∗^58)	c.301-10827_426+11057del (p.Met101_Lys142del)	c.1031del (p.Pro344Glnfs^∗^19)
Variant type	missense	missense	missense	missense	missense	missense	missense	missense	frameshift	a ∼22 kb deletion, including exon 2	frameshift
Zygosity, inheritance	het. *de novo*	het. *de novo*	het. *de novo*	het. *de novo*	het. *de novo*	het. *de novo*	het. *de novo*	het. *de novo*	het. *de novo*	het. *de novo*, het. inherited	het. *de novo*
Age at last visit (y, m)	5 y	13 y 1 m	13 y 10 m	19 y	1 y 5 m	7 y 5 m	12 y	3 y	newborn, 3 m	35 y, 2 y 9 m	1 y
Sex	female	female	male	male	male	female	female	male	female	male, male	male
Brain phenotype	microcephaly	macrocephaly	macrocephaly	macrocephaly with MRI abnormalities	macrocephaly, ventriculomegaly	extreme macrocephaly with Chiari malformation	borderline macrocephaly	microcephaly assumably caused by prenatal teratogens	intraventricular hemorrhage	no	no
Cognitive function	mild speech delay	moderate intellectual disability	speech delay	speech delay	motor delay	learning difficulty	moderate intellectual disability	profound developmental delay	N/A	no delay	no delay
Other findings/remarks	ectodermal dysplasia, CHD	no	sandal gap	cafe-au-lait spot, anxiety, hearing impairment, focal epilepsy	paroxysmal movements	choanal atresia, CHD	abnormal behaviors, Lennox-Gastaut syndrome	maternal drug consumption during early pregnancy	bilateral choanal atresia, CHD	adrenal insufficiency with no neurodevelopmental findings in both, CHD in son	nephrotic syndrome

CHD, congenital heart defect; het., heterozygous; m, months; N/A, not applicable; y, years.

Exome sequencing was performed on peripheral blood DNA using Agilent SureSelect Human All Exon V6 kit (Agilent Technologies, Santa Clara, CA, USA) on the Illumina 2500 system (Illumina, USA) for I-1, xGen Exome Research Panel IDT v1.0 kit (Integrated Device Technology, USA) on the Illumina Novaseq 6000 (Illumina, USA) platform for I-2, SureSelect Clinical Research Exome V2 kit (Agilent Technologies, Santa Clara, CA, USA) on an Illumina NextSeq 500 system (Illumina, USA) for I-4, Aligent SureSelect V4 kit (Agilent Technologies, Santa Clara, CA, USA) for I-6, the Ion AmpliSeq Library Kit 2.0 (Thermo Fisher Scientific, Waltham, MA, USA) on the Ion Proton sequencer (Thermo Fisher Scientific, Waltham, MA, USA) for I-7, the Roche KAPA Hyper Exome kit on the Illumina NextSeq 500 sequencing platform for I-9, the NimbleGen SeqCap EZ Library SR (Roche, Basel, Switzerland) on the Illumina NovaSeq 6000 platform (Illumina, San Diego, CA, USA) for I-10 and I-11. For the other four individuals, exome sequencing was performed with details not available.

### Structural modeling of protein variants

The structural effects of protein variants in the ectodomain were evaluated based on the crystal structure of the orthologous Xenopus znrf3 ectodomain in complex with Xenopus rspo2 (PDB: 4C9R^17^). The sequence identity between Xenopus and human proteins is 91% for the znrf3 ectodomain and 81% for rspo2. For all analyses, the human sequence numbering scheme was used throughout. For the interpretation of the variants located in the RING domain, a model of the ZNRF3 RING domain was retrieved from the Swiss-Model Repository.^18^ The complex with the E2 enzyme and ubiquitin was generated by adapting the binding mode from the complex of the orthologous Ark2C ring domain (PDB: 5D0M^19^). Missense variants were modeled using Swiss-Model,^20^ and alternative loop geometries were generated with ModLoop.^21^ The program RasMol^22^ was used for structure analysis and visualization.

### Cell-based transcription reporter assay

To evaluate the functional effects of the *ZNRF3* variants on the activity of the Wnt/β-catenin signaling, a SuperTopFlash (STF) transcriptional reporter assay was performed based on a protocol described previously.^23^ The wild-type *ZNRF3* cDNA in pcDNA3.1+C-HA (GenScript) was used to generate different *ZNRF3* variants by site-directed mutagenesis (SDM). SDM was performed using PfuUltra II Fusion HS DNA Polymerase (Agilent Technologies) followed by a template digestion with DpnI (New England BioLabs). Briefly, 2 × 10^4^ HEK293 cells (ATCC: CRL-1573) were plated per well in a 96-well plate. After 24 h, 8 ng of each *ZNRF3* cDNA construct, 40 ng pSTF (Addgene), and 2 ng pRenilla (Promega) plasmid were transiently transfected using FuGENE6 (Promega) with a FuGENE6 reagent:DNA ratio of 3:1. After 24 h of transfection, cells were replenished with a medium containing 20% of the conditioned medium from L Wnt3a cells (ATCC: CRL-2647), kindly provided by Prof. Gisou van der Goot (École Polytechnique Fédérale de Lausanne), and 100 ng/mL of the Wnt/β-catenin potentiator RSPO2 (PeproTech) and incubated for 24 h. Firefly and Renilla luminescence signals were detected using the Dual-Glo Luciferase Assay System (Promega). Firefly luminescence signals were normalized to their corresponding control Renilla luminescence signals.

## Results

### Characteristics of affected individuals and their genetic findings

**Figure 1:**
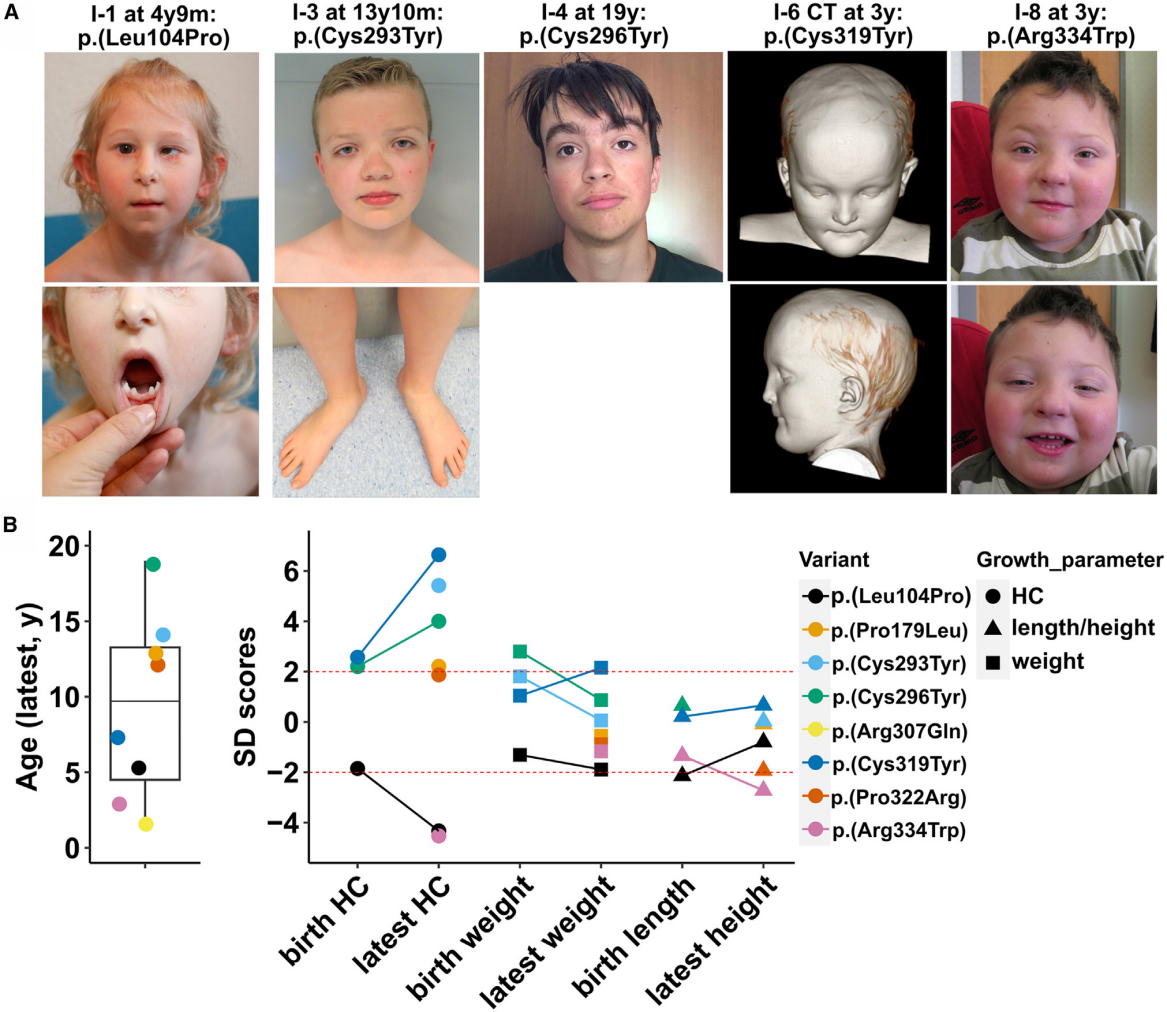
Affected individuals with *ZNRF3* missense variants (A) Photos of affected individuals. Individual 1 (I-1) at 4 years 9 months (p.Leu104Pro): note sparse hair, left-sided microphthalmia with the secretions around both eyes due to lacrimal duct obstruction, apparent hypotelorism, narrow nose and underdeveloped nares, apparently large protruding ears, deep philtrum, thin lip vermilion, oligodontia, and cone-shaped teeth. I-3 at 13 years 10 months (p.Cys293Tyr): note broad, high forehead, low-set ears, horizontal eyebrows, mild blepharoptosis, apparent hypertelorism, broad nasal tip, full lower lip vermillion, sandal gaps, and long toes. I-4 at 19 years (p.Cys296Tyr): note broad forehead, thick eyebrows, apparent hypertelorism, blepharoptosis, broad nasal tip, full lower lip vermillion, and low-set ears. I-6 CT at 3 years (p.Cys319Tyr): note broad and high, bulging forehead, narrow, down-slanting palpebral fissures (not shown), thin nose with broad nasal tip, thin upper lip, and short philtrum. I-8 at 3 years (p.Arg334Trp): flat facial profile, broad flat face, apparent hypertelorism, right epicanthic fold, narrow up-slanting palpebral fissures, pencil-thin eyebrows, broad nasal tip, smooth philtrum, thin upper lip, up-swept hair, and slightly up-turned earlobes. (B) Distribution of ages at last visit with a median of 9.7 years (range: 1.4–19) (left). Plotting of standard deviation (SD) scores of growth parameters at birth and latest visit for the affected individuals depicted with the corresponding missense variants shows two affected individuals with abnormally small head circumference (HC) and five affected individuals with abnormally large HC (right) (growth parameters for the affected individual with p.Arg307Gln were not available). CT, computed tomography; m, month; y, year.

**Figure 2:**
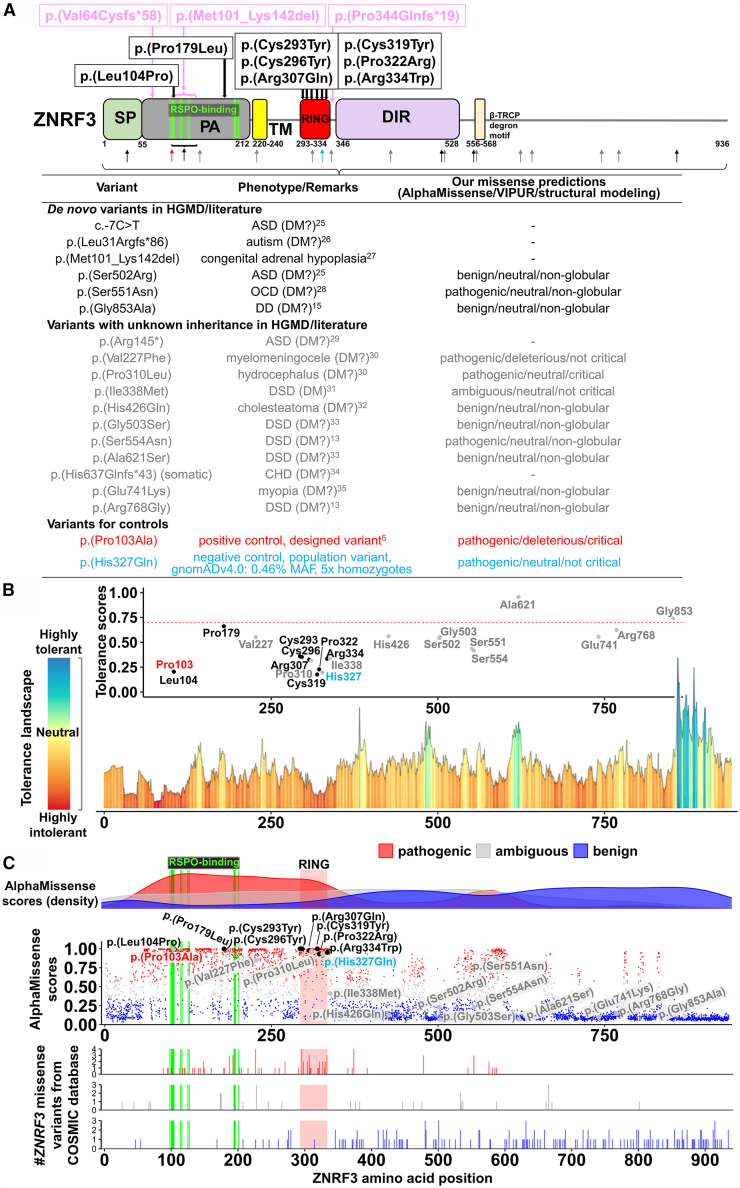
*ZNRF3* missense variants identified in affected individuals and their genetic landscape (A) Domain-specific clustering of variants identified in affected individuals (in black for *de novo* missense or in pink for frameshift or in-frame variants above the protein scheme). Domains include signal peptide (SP), protease associated (PA), which contains 14 RSPO binding sites (bright green), transmembrane (TM), RING-type E3 ligase (RING), disheveled (DVL) interacting region (DIR), and β-transducin repeats-containing a protein (β-TRCP) degron motif. Below the protein scheme, variants (black as *de novo*, or gray as unknown inheritance) reported in the Human Gene Mutation Database (HGMD) or literature are listed with associated phenotypes (HGMD classification: DM, disease-causing mutation; DM?, possible disease-causing mutation)^13^^,^^15^^,^^25^^,^^26^^,^^27^^,^^28^^,^^29^^,^^30^^,^^31^^,^^32^^,^^33^^,^^34^^,^^35^ and our various missense predictions. A designed variant (red letters and arrow) and a population variant (blue letters and arrow) are also shown, which were used in our study for positive and negative control, respectively. ASD, autism spectrum disorder; OCD, obsessive-compulsive disorder; DD, developmental disorder; DSD, disorder of sex development; CHD, congenital heart disease. (B) gnomAD-based tolerance landscape displays missense over synonymous ratios per position for the entire protein showing intolerance of variants with dark red for highly intolerant to dark blue for highly tolerant (inset, plotted with respective intolerance scores) identified in affected individuals (black letters) and in cases reported in HGMD/literature (gray letters). The designed variant and the population variant are shown in red and blue letters, respectively (data from Metadome). (C) Distribution of all possible missense variants in *ZNRF3* according to their AlphaMissense scores with the respective density plot (upper panel, blue dots, benign predictions; gray dots, ambiguous predictions; red dots, pathogenic predictions) shows enrichment of pathogenic predictions encompassing the RSPO binding sites to the RING domain and very high scores for pathogenic predictions for variants in affected individuals (black circles and letters) and the designed positive control variant p.Pro103Ala (red letters), while scores for variants reported in HGMD/literature are mostly benign (gray circles and letters). However, the common population variant p.His327Gln (blue letters) also showed a very high score for pathogenicity. RSPO-binding sites (green shaded area) and RING domains (red shaded area) are highlighted. Plotting of all *ZNRF3* missense variants reported in the Catalogue of Somatic Mutations in Cancer (COSMIC) database shows enrichment of pathogenic somatic variants in the RING domain as compared to the RSPO-binding sites (lower panel).

We identified eight individuals with *de novo* missense variants in *ZNRF3* (GRCh37 [RefSeq: NM_001206998.2]), all of whom showed brain phenotypes and harbored no additional obvious deleterious variants. Six of these had macrocephaly, while two presented with microcephaly (Figures 1A and 1B; Table 1). However, one of the latter had a history of heavy maternal drug and some alcohol consumption during pregnancy. All the affected individuals had developmental delay (8 of 8; 100%) with intellectual disability in 3 of 5, 60%, while some exhibited hypotonia (2 of 6; 33%), seizures (3 of 6; 50%), motor problems (3 of 8; 38%), or congenital heart defects (2 of 5; 40%) (Tables 1 and S1). Median age was 9.7 years (range 1.4–19) (Figure 1B; Table 1). One variant identified in the affected individual with microcephaly and unique ectodermal phenotypes is located in the RSPO-binding sites while six variants identified in the six affected individuals with macrocephaly are either located in the RING domain or in the PA domain (Figure 2A). The variant observed in the second individual with microcephaly is also located in the RING domain, but we assume that in this case, heavy prenatal exposure to opioids and other drugs are the likely cause of his microcephaly, which is in line with his growth restriction and preterm birth.^24^ Moreover, like the individuals with macrocephaly, he showed apparent hypertelorism. Therefore, the identified variants seem to cluster according to the associated phenotypes, thereby indicating a domain-specific genotype-phenotype correlation.

We collected four additional affected individuals with non-NDD phenotypes, including one harboring a *de novo* frameshift variant with heart defects, two harboring a large in-frame deletion (father *de novo* and son paternally inherited) with adrenal insufficiency, and one harboring a *de novo* frameshift variant with nephrotic syndrome (Table 1). Of note, the individual with congenital heart defect was born prematurely and experienced intraventricular hemorrhage, which is the likely cause of her mildly increased head circumference and hypotonia at birth and subsequent progressive microcephaly (I-9).

### Genetic landscape of our and reported *ZNRF3* variants

All *ZNRF3* missense variants identified in our study were unreported in the gnomAD control database (gnomAD v4.0.0, Table S1; Figure 2A), located in amino acid sequences that are (highly) intolerant toward missense variants (Figure 2B), and were predicted to be pathogenic by AlphaMissense (Figure 2C). The two truncating variants and one large in-frame deletion were also unreported in the gnomAD database (Table S1). *ZNRF3* is predicted to be haploinsufficient with an LOEUF (loss-of-function observed/expected upper bound fraction) score of 0.503 (recommended threshold for the loss-of-function [LoF] intolerant gene <0.6 in gnomAD v4.0.0), and the in-frame deletion affects an exon that is part of a functional domain (Figure 2A).

To date, there are several *ZNRF3* variants, especially missense, reported in the Human Gene Mutation database (HGMD) and literature for a variety of phenotypes,^13^^,^^15^^,^^25^^,^^26^^,^^27^^,^^28^^,^^29^^,^^30^^,^^31^^,^^32^^,^^33^^,^^34^^,^^35^ most of which lacked information about mode of inheritance as well as support for pathogenicity (Figure 2A). Assessment by our missense predictions using several tools including AlphaMissense,^36^ VIPUR,^37^ and manual structural modeling revealed no consistent evidence for pathogenicity for almost all of these missense variants. Nonetheless, despite unknown inheritance, one variant c.929C>T (GenBank: NM_001206998.2) (p.Pro310Leu) observed in an individual with hydrocephaly could probably be pathogenic since according to our structural modeling, the proline stabilizes a beta turn that is located near the zinc finger, which could ultimately lead to disrupted ligase activity, which would be in line with our suggested genotype-phenotype correlation.

As *ZNRF3* is a tumor repressor gene and has been shown to be frequently mutated in various types of cancer, we collected all *ZNRF3* missense variants reported in the Catalogue Of Somatic Mutations In Cancer (COSMIC) database and found an enrichment of pathogenic somatic variants in the RING domain, two of which are the same as the germline variants observed in our cohort (c.536C>T [GenBank: NM_001206998.2] [p.Pro179Leu] and c.920G>A [GenBank: NM_001206998.2] [p.Arg307Gln]) as compared to those in the RSPO-binding sites (Figure 2C; *p* = 0.0006 [Fischer’s exact test]), suggesting a possible increased risk for cancer in affected individuals with macrocephalic NDD harboring deleterious variants in the RING domain. In addition, we observed two variants from the COSMIC database, which are in the RSPO-binding sites, of which only one (c.302T>C [GenBank: NM_001206998.2] [p.Met101Thr]) was predicted by AlphaMissense to be pathogenic and associated with endometrioid carcinoma. The COSMIC variants in the RING domain that were predicted by AlphaMissense to be pathogenic were observed in a variety of cancers, including endometrioid carcinoma. Therefore, we observed no enrichment for certain cancer type for either variants in the RSPO-binding or RING domain.

### Functional effects of overexpressing *ZNRF3* variants on the Wnt/β-catenin signaling

**Figure 3:**
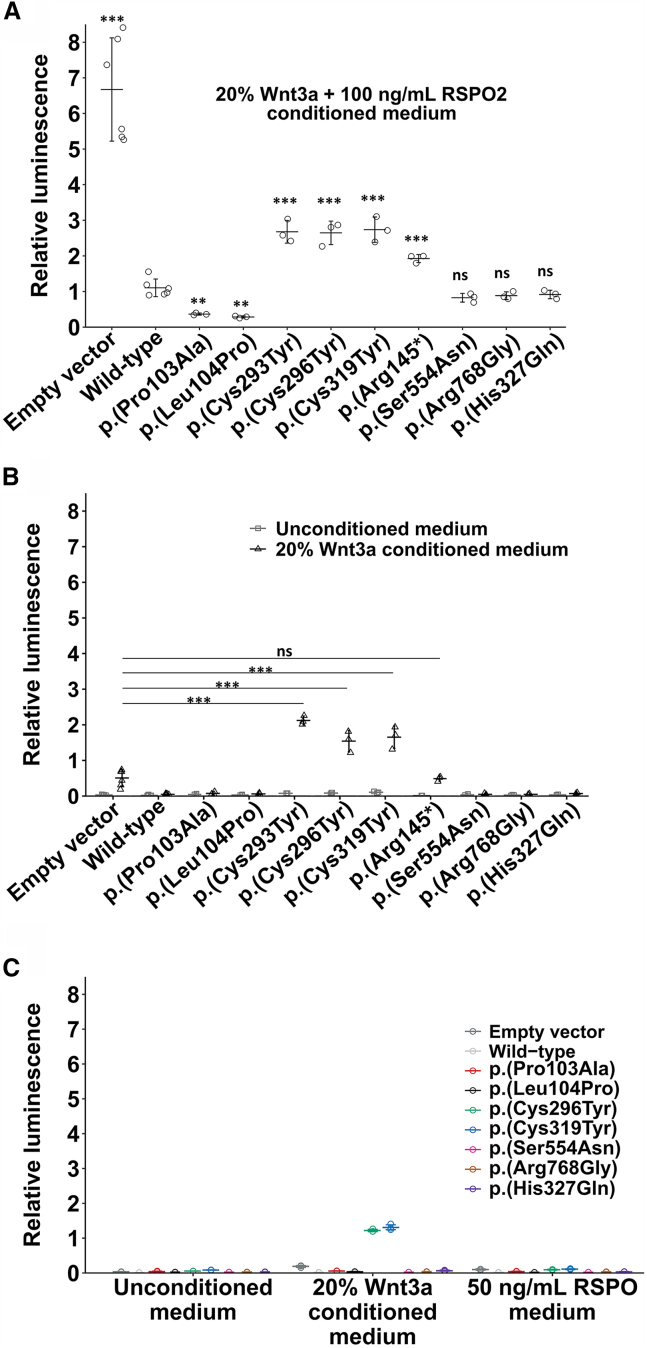
Effects of overexpressing *ZNRF3* variants on Wnt/β-catenin signaling Wnt/β-catenin signaling was measured using a luciferase-based transcription reporter assay known as SuperTopFlash (STF) assay. HEK293 cells were each transfected with plasmids containing either wild-type or different *ZNRF3* cDNA variants, STF reporter, and Renilla control plasmids. (A) STF activity upon dual activation with Wnt ligand Wnt3a and potentiator RSPO. The variants p.Leu104Pro and positive control p.Pro103Ala, which were derived from the microcephaly-affected individual and designed in a study on cancer aspects of *ZNRF3*,^38^ respectively, showed an attenuated STF activity, while the variants p.Cys293Tyr, p.Cys296Tyr, and p.Cys319Tyr from the macrocephaly-affected individuals exhibited an enhanced STF signal. In addition, STF activity of the variants p.Ser554Asn and p.Arg768Gly from a previous study claiming their association with human sex reversal, as well as the population variant p.His327Gln, was unchanged compared to the wild-type control variant. Results were from three independent experiments (mean ± SD). ^∗∗^*p* < 0.01; ^∗∗∗^*p* < 0.001; ns, not significant (ANOVA followed by the Dunnet’s test). (B) STF activity upon activation with Wnt ligand Wnt3a only. Overall, low levels of basal STF activity were slightly increased by the addition of 20% Wnt3a conditioned medium. However, Wnt3a-induced STF activity for macrocephaly variants was highly increased even as compared to the empty vector control. Results were from three independent experiments (mean ± SD). ^∗∗∗^*p* < 0.001 (ANOVA followed by the Dunnet’s test). (C) STF activity upon activation with Wnt ligand Wnt3a or Wnt potentiator RSPO only. Results were from one experiment. Error bars show standard deviation from three technical replicates.

We investigated the effects of overexpressing selected *ZNRF3* variants on Wnt/β-catenin signaling using an *in vitro* transcriptional reporter assay called STF assay. We included a missense variant, c.307C>G (GenBank: NM_001206998.2) (p.Pro103Ala), as a positive control, which was designed and functionally tested to be deleterious by a previous study,^38^ and a population variant, c.981C>A (GenBank: NM_001206998.2) (p.His327Gln), as a negative control, which is relatively frequently observed in the gnomAD control database (rs55775472, gnomAD v4.0.0: 7,182 of 1,614,178 alleles examined with 42 homozygotes reported) but is in the RING domain and has a high AlphaMissense score (0.9) (Figure 3A). To obtain a range of STF activity that reliably reflects changes in the Wnt/β-catenin signaling levels, we synergistically induced the STF activity by simultaneously adding the Wnt/β-catenin signaling ligand Wnt3a and the Wnt/β-catenin signaling potentiator RSPO2, which has the highest affinity to ZNRF3 compared to other RSPO proteins.^17^ We found that in comparison to the induced STF activity for an empty control vector, the STF activity by overexpressing the wild-type ZNRF3 was strongly reduced (Figure 3A). We observed that this STF activity was further reduced by our c.311T>C (GenBank: NM_001206998.2) (p.Leu104Pro) variant, as well as the positive control p.Pro103Ala variant, but relatively strongly enhanced by our three other variants, c.878G>A (GenBank: NM_001206998.2) (p.Cys293Tyr), c.887G>A (GenBank: NM_001206998.2) (p.Cys296Tyr), and c.956G>A (GenBank: NM_001206998.2) (p.Cys319Tyr). In contrast, overexpression of the population variant p.His327Gln resulted in no change in the STF activity. We also tested two additional variants, c.1661G>A (GenBank: NM_001206998.2) (p.Ser554Asn) and c.2302A>G (GenBank: NM_001206998.2) (p.Arg768Gly), obtained from a previous study, which were identified in individuals with disorders of sex development,^13^ but found no STF activity change. We also accessed a truncating variant, c.433C>T (GenBank: NM_001206998.2) (p.Arg145^∗^), and found that it showed the same result as the empty vector control upon activation with Wnt3a alone (Figure 3B). As this nonsense variant could not undergo nonsense-mediated decay, as it is a plasmid-derived cDNA sequence and lacked half of the ectodomain and full transmembrane and intracellular domains, it could not function on its own unless as a dimer with the endogenous wild type. The dimerization might hold true as we observed its STF activity higher than that for the exogenous wild type but lower than that for the RING variants upon activation with Wnt3a and RSPO (Figure 3A).

Upon activation of Wnt/β-catenin signaling by Wnt3a alone, we observed a profoundly enhanced STF activity for the RING variants compared to the wild type as expected, as disruption of the RING ligase activity by the variants in the RING domain compromised the inhibition of the Wnt/β-catenin signaling by ZNRF3 (Figure 3B). This STF activity was also higher than that for the empty control where the STF activity was a baseline level of the inhibition by endogenous ZNRF3. As ZNRF3 forms a dimer to function as a regulator of Wnt/β-catenin signaling, this phenomenon suggests that the exogenous RING variants could also form dimers with the endogenous wild-type ZNRF3 and hampered the overall inhibitory function of ZNRF3, and hence the observed STF activity was even higher than that for the empty control, which altogether suggests a dominant negative effect of such deleterious variants in the RING domain of ZNRF3. This finding is in line with the STF activity for the RING variants that was unchanged upon activation with RSPO alone (Figure 3C).

### Structural modeling of ZNRF3 protein variants

**Figure 4:**
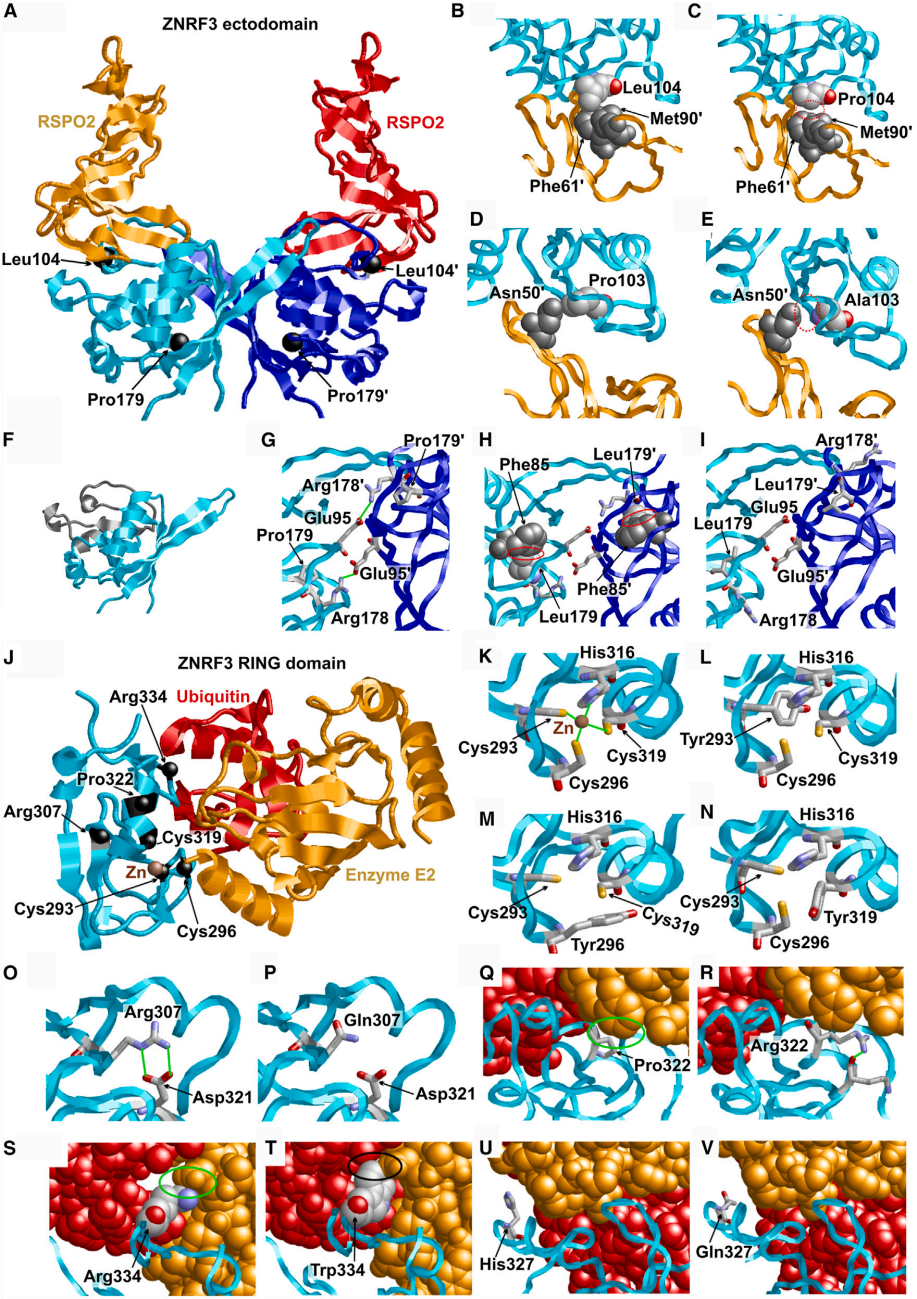
Structural modeling of the protein variants located in the ZNRF3 ectodomain or RING domain (A) Structure of the ZNRF3 ectodomain homodimer (cyan and blue) in complex with RSPO2 (orange and red). The position of Leu104 and Pro179 is indicated by black balls and are labeled. Primes are used to distinguish residues from different subunits. (B and C) Effect of the p.Leu104Pro exchange. (B) Leu104 forms tight interactions with Phe61 and Met90 of RSPO2. (C) In the p.Leu104Pro variant, these interactions are lost (red dotted oval), resulting in weaker RSPO2 binding. (D and E) Structure of the ZNRF3-RSPO2 heterodimer (cyan and orange) highlighting the effect of the p.Pro103Ala exchange. (D) Pro103 forms tight interactions with Asn50 of RSPO2. (E) In the p.Pro103Ala variant, these interactions are lost (red dotted oval) resulting in weaker RSPO2 binding. (F) The p.Met101_Lys142 deletion (gray) removes large parts of the ectodomain thereby disrupting the domain structure. (G–I) Effect of the p.Pro179Leu exchange. (G) In the Pro179 wild type, the adjacent Arg178 forms a salt bridge (green line) with Glu95 in the second subunit of the ectodomain homodimer. (H) The presence of a leucine at position 179 causes steric clashes with Phe85 (red circles). (I) Altered conformation of the loop around Leu179 that avoids steric clashes with Phe85. In this conformation, Arg178 can no longer interact with Glu95 thereby disrupting the Arg178-Glu95 salt bridge. (J) Structure of the ZNRF3 RING domain (cyan) in complex with enzyme E2 (orange) and ubiquitin (red). The position of the residues, for which mutations were observed, are indicated by black balls and are labeled. (K) Enlargement of one zinc finger in wild-type ZNRF3 in which the zinc ion (brown) is tetrahedrally coordinated by three cysteines and one histidine (zinc interactions highlighted in green). (L–N) Mutations of the zinc coordinating cysteines (L) p.Cys293Tyr, (M) p.Cys296Tyr, (N) p.Cys319Tyr lead to a loss of the zinc ion resulting in domain destabilization. (O and P) Effect of the p.Arg307Gln exchange. (O) Arg307 forms a salt bridge with Asp321, which cannot be formed by the shorter and uncharged sidechain in the (P) Gln307 variant. (Q and R) Effect of the p.Pro322Arg exchange. (Q) Pro322 forms favorable interactions with the E2 enzyme (green oval), whereas (R) Arg322 rather forms interactions within the RING domain (green line). (S and T) Effect of the p.Arg334Trp exchange. (S) Arg334 forms favorable interactions with the E2 enzyme (green oval), whereas (T) Trp334 causes steric clashes with E2 (black circle) thereby hampering the function of the RING-domain. (U) His327 is located close to the interface with the E2 enzyme but does not form intermolecular interactions in the model. (V) The Gln327 variant exhibits rather similar structural properties as the wild-type complex.

To better understand the underlying mechanisms, we performed structural modeling of the ZNRF3 protein variants. Three of the variants detected in the present study (p.Leu104Pro, p.Pro179Leu, NC_000022.10[NM_001206998.2]:c.301_10827_426+11057del [p.Met101_Lys142del]) are located in the ectodomain of ZNRF3, which forms a homodimer and binds to RSPOs (Figure 4A). RSPOs are secreted agonists of Wnt signaling in vertebrates. Leu104 is located in the ectodomain-RSPO2 heterodimer interface (Figure 4A), and Leu104 forms hydrophobic interactions with Phe61 and Met90 of RSPO2 (Figure 4B). These interactions cannot be formed by the compact cyclic sidechain of Pro104 (Figure 4C), thereby weakening the ZNRF3-RSPO interaction. This will prevent the potentiation by RSPO, thereby enhancing the negative feedback and attenuating the Wnt signaling, which is in line with the decreased STF activity of the p.Leu104Pro variant observed in our functional assay. Pro103 is next to Leu104 and forms tight interactions with Asn50 of RSPO2 (Figure 4D). In the p.Pro103Ala variant, which was designed and functionally tested by a previous study on cancer aspects to decrease the Wnt signaling,^38^ these interactions are lost, resulting in weaker RSPO2 binding (Figure 4E), and we also observed a decreased Wnt signaling in our functional assay like in the p.Leu104Pro variant. These findings substantiate the link between disruption of RSPO-binding and attenuated Wnt signaling.

In the p.Met101_Lys142del variant, large parts of the ectodomain are missing (Figure 4F), which is expected to disrupt the entire domain structure thereby leading to a loss of homodimerization and RSPO-binding. As the two affected individuals with this deletion did not show NDD with brain phenotypes, such loss of homodimerization and RSPO-binding in the heterozygous state may have other functional consequences, warranting further investigations.

Pro179 is located close to the homodimer interface (Figure 4A), and the adjacent residue Arg178 forms a salt bridge with Glu95 of the second subunit (Figure 4G). The p.Pro179Leu exchange causes steric clashes between Leu179 and Phe85 (Figure 4H), leading to an altered conformation of the loop around Leu179 in which Arg178 can no longer interact with Glu95 (Figure 4I), which hampers homodimer formation. That means that p.Pro179Leu has a significantly different structural and functional effect compared to p.Leu104Pro, although both variants are located in the ectodomain. Therefore, an increase in Wnt signaling in a cell-based transcriptional reporter assay may be assumed for the p.Pro179Leu variant, in contrast to a decrease in Wnt signaling observed for the p.Leu104Pro variant.

The remaining sequence variants detected in the present study are in the RING domain of ZNRF3, which is expected to interact both with ubiquitin and the ubiquitin-conjugating enzyme E2 (Figure 4J) based on the crystal structure of the orthologous Ark2C RING domain.^19^

The ZNRF3 RING domain contains two zinc fingers. Cys293, Cys296, and Cys319 belong to the same zinc finger and are involved in the tetrahedral coordination of one zinc ion (Figure 4K). Since zinc fingers can only be formed by cysteines or histidines, exchange of either Cys293, Cys296, or Cys319 by tyrosine will disrupt the tetrahedral zinc coordination and hence lead to a loss of the zinc ion (Figures 4L–4N). This loss is expected to cause unfolding of the RING domain, which will also hamper or even completely impede its interaction with E2 enzymes. This will disrupt the ligase activity, compromise the negative feedback, and thereby enhance the Wnt signaling, which is in line with the increased STF activity of the three variants observed in our functional experiments.

Arg307 forms an intramolecular salt bridge to Asp321, which is disrupted in the p.Arg307Gln variant (Figures 4O and 4P). Therefore, this variant is also expected to destabilize the RING domain in a similar fashion to the Cys-to-Tyr variants above. Pro322 and Arg334 are, according to orthologous complexes, in the interface between ZNRF3 and E2 enzymes (Figures 4Q and 4S). The c.965C>G (GenBank: NM_001206998.2) (p.Pro322Arg) and c.1000C>T (GenBank: NM_001206998.2) (p.Arg334Trp) exchanges lead to unfavorable interactions at the interface (Figures 4R and 4T) and are therefore predicted to reduce the affinity between ZNRF3 and E2 enzyme. Although the sites of both variants are spatially close to the population variant p.His327Gln, molecular modeling did not reveal any negative structural effects of the His327Gln exchange (Figures 4U and 4V). As the p.Arg307Gln, p.Pro322Arg, and p.Arg334Trp variants were predicted to disrupt interactions with the E2 enzyme like the p.Cys293Tyr, p.Cys296Tyr, and p.Cys319Tyr variants (Figure 3), for which increased Wnt signaling in a cell-based transcriptional reporter assay was observed, the same effect on Wnt signaling may as well be assumed for such variants.

As opposed to the decreased Wnt signaling apparently observed for the variants (p.Leu104Pro and p.Pro103Ala) that disrupt the RSPO-binding sites only, the increased Wnt signaling were observed or predicted by our modeling for deleterious variants in the RING domain (p.Cys293Tyr, p.Cys296Tyr, p.Cys319Tyr, p.Arg307Gln, p.Pro322Arg, and p.Arg334Trp) and for the deleterious variant p.Pro179Leu in the protease-associated domain, reflecting a higher prevalence of macrocephaly than that of microcephaly in our cohort.

The three variants p.Ser554Asn, p.Arg768Gly, and c.1861G>T (GenBank: NM_001206998.2) (p.Ala621Ser), which were described by Harris et al.,^13^ in association with disorders of sexual development are in a nonglobular region and are therefore predicted to have no critical effect on the protein structure.

## Discussion

**Figure 5:**
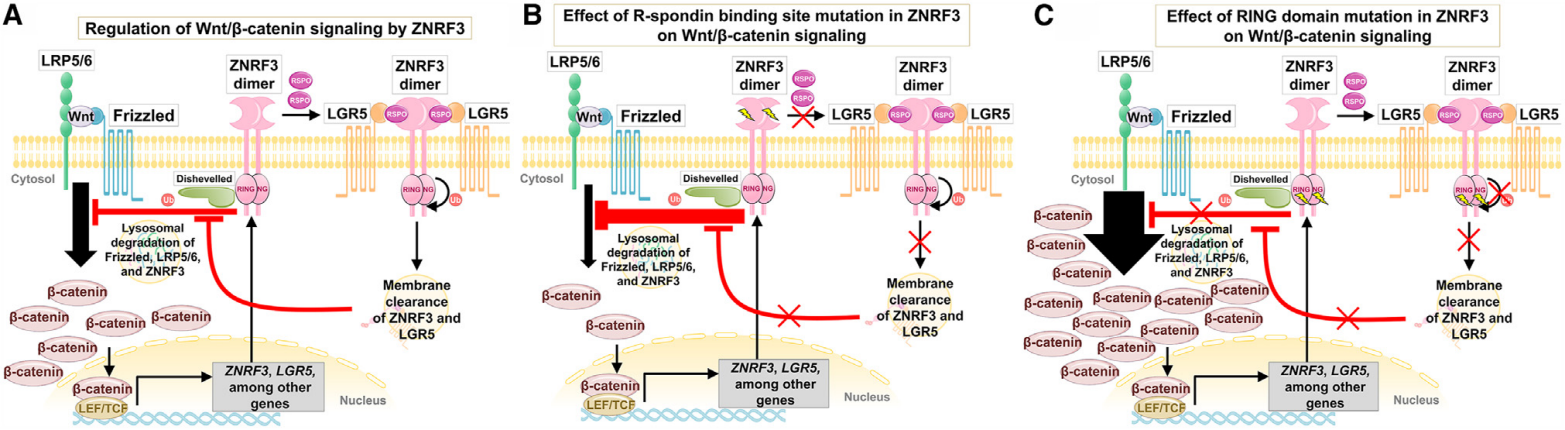
Simplified schematic representation of the regulation of Wnt/β-catenin signaling and the effects of ZNRF3 mutants (A) Binding of Wnt ligand to its receptor frizzled, and LRP5/6 leads to a series of events (not depicted) that result in an accumulation of β-catenin, which in turn binds to LEF/TCF (lymphoid enhancer factor/T cell factor) transcription factor and induces expression of a variety of genes important for development and homeostasis. Wnt/β-catenin signaling also induces the expression of several regulators of the pathway itself, e.g., ZNRF3 and LGR5. ZNRF3 is an E3 ubiquitin ligase that can be recruited as a dimer to the Wnt receptor complex through interaction with disheveled to mediate the transfer of ubiquitin to frizzled. The ubiquitinated Wnt receptor complex is then internalized and subjected to lysosomal degradation, resulting in a negative feedback regulation of the Wnt/β-catenin signaling. This can be counteracted by another Wnt modulator, R-spondin (RSPO), which binds to both ZNRF3 and a coreceptor, LGR5, to induce their internalization probably via ZNRF3-mediated autoubiquitination, resulting in potentiation of Wnt/β-catenin signaling as depicted. (B) ZNRF3 mutations within the binding site to RSPO that disrupt the interaction of both proteins abolish the RSPO-mediated potentiation, thus leading to a decreased Wnt/β-catenin signaling. (C) ZNRF3 mutations in the RING domain that impair its ligase activity abrogate the ability of ZNRF3 to induce ubiquitination and promote degradation of both frizzled and ZNRF3, leading to an increased Wnt/β-catenin signaling. Ub, ubiquitin. Yellow lightning bolt represents mutation.

We have identified *de novo* deleterious missense variants in the gene *ZNRF3* in affected individuals with mirror brain phenotypes: microcephaly and macrocephaly. These variants affected two distinct domains of the ZNRF3 protein and, as predicted by our comprehensive structural modeling, exert distinct impacts on the ZNRF3 activity that ultimately resulted in two opposite functional consequences in the Wnt/β-catenin signaling. The microcephaly variant p.Leu104Pro, as well as the designed positive control p.Pro103Ala, were predicted to disrupt the binding affinity to RSPO, and we demonstrated that they attenuate Wnt/β-catenin signaling. Conversely, all macrocephaly variants were predicted to disrupt the ubiquitination activity of the ZNRF3 protein and we indeed showed representatively for p.Cys293Tyr, p.Cys296Tyr, and p.Cys319Tyr enhanced Wnt/β-catenin signaling in a dominant-negative manner. According to these findings, we describe a mechanistic scheme of the functional defect-specific and opposing effects of *ZNRF3* variants on Wnt/β-catenin signaling (Figures 5A, 5B, and 5C). In contrast, truncating/large in-frame deletion variants were not associated with NDD phenotypes, remained variants of uncertain significance (VUSs), and may be associated with distinct conditions through haploinsufficiency or other mechanisms.

### Effects of dysregulated Wnt/β-catenin signaling on human brain size

Wnt/β-catenin signaling has a role in neural progenitor proliferation and neuronal differentiation.^39^ It has been shown that decreased Wnt/β-catenin signaling leads to decreased proliferation of the neural progenitor cells (NPCs), whereas its increase expands the size of the neural progenitor pool.^40^^,^^41^ In line with this, proper size control of the neural progenitor pool has been frequently referred to as an underlying event for the assembly of the correct brain volume,^40^^,^^42^^,^^43^ where NPCs have to undergo a rapid cell proliferation to generate a proper cell amount before switching to neuronal differentiation.^44^^,^^45^ Moreover, mutations in several established microcephaly genes, such as *ASPM* (MIM: 605481),^46^ *CDK5RAP2* (MIM: 608201),^47^ and *CENPJ* (MIM: 609279),^48^ are known to likely disrupt such control, disturb the balance between proliferation and differentiation of the NPCs, and subsequently decrease the neural progenitor pool size. On the other hand, mutations in the macrocephaly genes *PTEN* (MIM: 601728)^49^ and *CHD8* (MIM: 610528)^50^ are causatively linked to excessive proliferation of NPCs. Nevertheless, the Wnt signaling pathway is very complex, and there are 233 genes annotated for “Wnt signaling pathway” in the Gene Ontology Biological Process (Table S2). Of these, 16 (∼7%) genes are linked to microcephaly (Tables 2 and S2) while 17 (∼7%) genes are linked to macrocephaly, according to the Online Mendelian Inheritance in Man (OMIM) (Tables 2 and S2). Among them, one gene, *RAC1* (MIM: 602048), whose function is more established for the noncanonical Wnt signaling pathway, has been linked to NDD with variable head circumference (micro-, normo-, or macrocephaly) and *de novo* missense variants without a distinct clustering pattern and a clear underlying mechanism.^51^^,^^52^ Among the ∼700 β-catenin physical target genes as measured in human embryonic stem cells,^53^ next to *ZNRF3*, 19 genes negatively regulate the canonical Wnt signaling (Tables 2 and S3). From these genes, four are associated either with microcephaly (*CUL3* [MIM: 603136]) or macrocephaly (*CDH2* [MIM: 114020], *GLI3* [MIM: 165240], and *WNT5A* [MIM: 164975]) (Tables 2 and S3). Among the other 15 genes, six are described for NDD without micro- or macrocephaly (Tables 2 and S3) while nine are not yet established for NDD phenotypes in OMIM or the SysNDD database. Among the nine genes, *AXIN2* (MIM: 604025) is established for oligodontia while *DKK1* (MIM: 605189) and *FOXO1* (MIM: 136533) are tentatively linked to NDD (Table 2). *Dkk1* has been shown to modulate Wnt activity during head morphogenesis in mice^54^ in which a *de novo* deleterious missense variant, p.Arg120Leu, has been identified in two unrelated individuals with Chiari malformation type I (MIM: 118420) and, in one of them, hydrocephalus,^55^ phenotypes also observed in one (I-6, Table 1) of the affected individuals with macrocephaly. This correlation is further substantiated by a study showing that another Wnt negative regulator, FOXO1, can cause Chiari malformation, which was observed in a child with lambdoid craniosynostosis.^56^ In addition, a well-known homolog of ZNRF3, ring finger protein 43 (RNF43 [MIM: 612482]) with an ectodomain protein sequence identity of 39% to ZNRF3,^17^ is also negatively regulating Wnt signaling^57^ and is frequently mutated in several types of cancer^58^ but has not yet been causally linked to NDD.

**Table 2:** Summary of genes involved in Wnt signaling pathway and Wnt target genes stratified according to brain size or respective phenotypes based on the OMIM clinical synopsis, SysNDD, or HGMD entries

**Gene**	**MIM**	**Brain size or respective phenotype**^a^
**Wnt signaling pathway genes**		

*CDC42*	116952	microcephaly
*CSNK2A1*	115440	microcephaly
*CTNNB1*	116806	microcephaly
*CUL3*	603136	microcephaly
*LRP5*	603506	microcephaly
*MACF1*	608271	microcephaly
*MESD*	607783	microcephaly
*PORCN*	300651	microcephaly
*RAC1*	602048	microcephaly or macrocephaly
*SHH*	600725	microcephaly
*SMO*	601500	microcephaly
*SOX4*	184430	microcephaly
*TBL1XR1*	608628	microcephaly
*VRK1*	602168	microcephaly
*WLS*	611514	microcephaly
*WWOX*	605131	microcephaly
*AMER1*	300647	macrocephaly
*APC2*	612034	macrocephaly
*AXIN1*	603816	macrocephaly
*CHD8*	610528	macrocephaly
*CPE*	114855	macrocephaly
*DVL1*	601365	macrocephaly
*DVL3*	601368	macrocephaly
*FBXW11*	605651	macrocephaly
*MED12*	300188	macrocephaly
*MITF*	156845	macrocephaly
*NXN*	612895	macrocephaly
*PTEN*	601728	macrocephaly
*ROR2*	602337	macrocephaly
*SOST*	605740	macrocephaly
*TBL1X*	300196	macrocephaly
*WNT5A*	164975	macrocephaly

**β-catenin physical target genes that encode Wnt negative regulators**		

*CUL3*	603136	microcephaly
*CDH2*	114020	macrocephaly
*GLI3*	165240	macrocephaly
*WNT5A*	164975	macrocephaly
*BMP2*	112261	other NDD
*DACT1*	607861	other NDD
*NPHP4*	607215	other NDD
*PRICKLE1*	608500	other NDD
*TCF7L2*^b^^,^^c^	602228	other NDD
*WNT4*	603490	other NDD
*DKK1*^55^	605189	tentative NDD
*FOXO1*^c^	136533	tentative NDD
*AXIN2*	604025	oligodontia
*DAB2IP*	609205	no established phenotype
*FZD1*	603408	no established phenotype
*KREMEN2*	609899	no established phenotype
*MLLT3*	159558	no established phenotype
*NKD1*	607851	no established phenotype
*PSMA6*	602855	no established phenotype
*ZNRF3*	612062	no established phenotype

NDD, neurodevelopmental disorder.

a Phenotype based on the OMIM clinical synopsis if not otherwise indicated.

b Described for “definitive” NDD based on the SysNDD entries.

c Described for NDD based on the HGMD entries.

**Figure 6:**
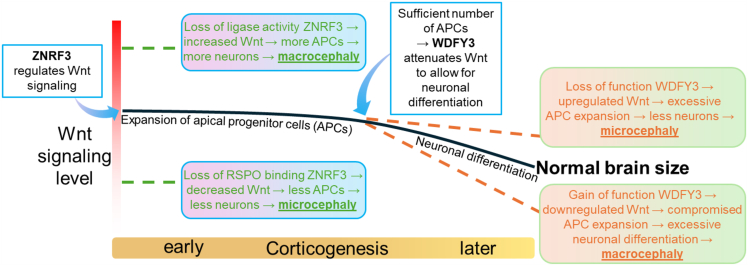
A simplified summary of the possible effects of deleterious variants in *ZNRF3* vs. *WDFY3* on Wnt signaling during corticogenesis Corticogenesis is tightly regulated by Wnt signaling level, which is in turn regulated by *ZNRF3* and, tentatively, *WDFY3*, albeit at different time points. At early stages of corticogenesis, Wnt is activated for the expansion of the apical progenitor cells (APCs), which induce the expression of *ZNRF3* that functions as a negative feedback regulator of the Wnt signaling. When the number of APCs is sufficient, *WDFY3* starts to attenuate Wnt signaling to allow for neuronal differentiation. Disrupting variants in *ZNRF3* or *WDFY3*, which act at early or later stages of corticogenesis, respectively, can lead to opposing brain size phenotypes. Graphs are shown with approximate scaling.

Although the role of *WDFY3* in Wnt signaling pathway is still obscure so that the reported observations can be a result of distant downstream events, it is thus far the only (tentative) canonical Wnt signaling pathway gene that is assumably associated with two opposing phenotypes of the brain size,^3^ which merits addressing here. The underlying mechanism for these phenotypes is unclear and is apparently different from our proposed mechanisms. In fact, the authors of that study^3^ attributed upregulation of Wnt/β-catenin signaling to microcephaly and downregulation to macrocephaly. Since our missense variants led to loss of function of two distinct domains (RSPO binding site and RING ligase domain), their mechanisms of action resulted in opposite effects on Wnt/β-catenin signaling. Unlike *WDFY3*-associated macrocephaly where Wnt/β-catenin signaling was downregulated, Wnt/β-catenin signaling was enhanced for missense variants in *ZNRF3* from the macrocephaly affected individuals, while decreased for the *ZNRF3* missense variant from the microcephaly affected individual. Given that the activity of *WDFY3* starts later than that of *ZNRF3*, it is likely that the *WDFY3* missense variant that was associated with microcephaly and led to upregulation of Wnt/β-catenin signaling^3^ acts through excessive clonal expansion of apical progenitors but compromised neuronal differentiation as a plausible pathomechanism^59^ (Figure 6). Furthermore, almost all their *WDFY3* variants causing macrocephaly were truncating, suggesting *WDFY3* haploinsufficiency as a pathomechanism for macrocephaly. In contrast, truncating variants in *ZNRF3* in our cohort, which remain VUS were identified in individuals with non-NDD phenotypes, while our pathogenic missense variants in *ZNRF3* were associated with micro- or macrocephalic developmental disorders.

This phenomenon seems to be attributable to the dynamics of the Wnt/β-catenin signaling during the embryonic development of the human brain where it peaks in the beginning of corticogenesis and decreases toward the later stages thereof^1^ (see Figure 6). It is indeed during the later stages of corticogenesis, during which once the number of apical progenitors is sufficient, WDFY3 starts to downregulate the Wnt/β-catenin signaling for neuronal differentiation to ultimately achieve proper brain size.^59^ At this stage, LoF WDFY3 will increase Wnt signaling and enhance symmetric cell division of apical progenitor cells (APCs) and hamper asymmetric cell division so that less neurons are produced, which will eventually lead to microcephaly. In contrast, gain-of-function WDFY3 will abnormally decrease Wnt signaling and attenuate symmetric but enhance asymmetric cell divisions so that more neurons will be generated, which eventually leads to macrocephaly. In accordance, *WDFY3* haploinsufficiency seemed to be compensated by an enhanced ubiquitination and proteosome-dependent degradation of DVL,^3^ leading to uncontrolled downregulation of Wnt/β-catenin signaling, enhanced neurogenesis, and hence macrocephaly.^60^ As ZNRF3 is a negative feedback regulator of Wnt/β-catenin signaling,^5^ it is expressed when Wnt pathway is active. Therefore, disruption of ZNRF3 activity predominantly affects the early stage of corticogenesis where Wnt/β-catenin pathway is highly active. At this stage, APCs undergo rapid symmetrical cell division to reach a sufficient number. Therefore, if there is loss of Wnt feedback regulation from ZNRF3 due to mutations in the RING domain, there will be overproduction of APCs, which leads to increased number of neurons and eventually macrocephaly (Figure 6). On the other hand, if binding to RSPO is abrogated, ZNRF3 feedback activity will be enhanced and Wnt signaling decreased, which leads to reduced numbers of APCs, neurons, and eventually microcephaly (see Figure 6). Accordingly, the *ZNRF3* variants from the affected individuals with opposite brain size phenotypes described in our study linked increased Wnt/β-catenin signaling to macrocephaly and decreased Wnt/β-catenin signaling to microcephaly.

While ZNRF3 has been mainly studied on its function in the Wnt signaling pathway, some studies have suggested its role in other signaling cascades. It has been shown that znrf3 can be triggered by rspo2 to internalize and degrade the type I bone morphogenetic protein (BMP) receptor bmpr1a during the regulation of dorsoventral axis formation in *Xenopus*.^61^ Very recently, a study has reported the regulatory function of ZNRF3 for the epidermal growth factor receptor in cancer cell lines.^62^ As these other functions of ZNRF3 are still not well established and the extent of crosstalk to its Wnt-related function is not yet well investigated, their role for the observed brain and other phenotypes remain open.

### Dominant-negative effects of *ZNRF3* variants from individuals with macrocephaly

ZNRF3 has been shown to have a strong propensity to dimerize.^17^^,^^63^ Thus, it is likely that in our STF assay dimerization occurred among the plasmid-derived ZNRF3 variants and the endogenous wild-type ZNRF3. Upon activation with Wnt3a alone, STF activity for the empty vector control reflects the Wnt signaling level that was inhibited and maintained by the endogenous wild-type ZNRF3. As expected, the STF activity was decreased upon the addition of the exogenous wild-type ZNRF3. The STF activity for the variants in the RING domain was theoretically expected to increase back to the level of the empty vector control, or the nonsense variant p.Arg145^∗^, due to disrupted RING ligase activity, but the increase was much higher than that of the empty vector control. This suggests that the variants in the RING domain negatively interfere and act dominantly with the activity of the endogenous wild-type ZNRF3, assumably through dimerization. Yet, upon addition of RSPO, the effect of such RING domain ZNRF3 variants can still be further modulated as their RSPO binding site is unaffected. Therefore, the dominant negative effect of these variants is compromised as evident in their STF activity being lower than that of the empty vector control (Figure 3A).

### Further phenotypic aspects of *ZNRF3* variants

With regards to the relationship between *ZNRF3* variants and a human phenotype of disorder of sex development in the previous study,^13^ we could not reproduce the effects for their variants. To gain more insight, we have attempted to perform structural modeling for these variants but were hampered as they were outside any ZNRF3 protein domain. However, we speculated that the discrepancy might be partly explained by the condition used for the STF assay in the previous study, without adding Wnt3a and RSPO to achieve a reliable range of STF activity. Therefore, based on our own assessment, we cannot support the claimed association between the reported *ZNRF3* variants and sex reversal.

In contrast, individuals with truncating or large in-frame deletion variants in our cohort had non-NDD phenotypes. Although we consider these variants candidates for causing adrenal insufficiency, congenital heart defects, and nephrotic syndrome, they remain VUS until further proof, but at least suggests that haploinsufficiency of *ZNRF3* is not causing neurodevelopmental problems.

It has been shown that only 14 amino acids in the ectodomain, for which almost any exchange is predicted to be pathogenic by AlphaMissense, are involved in RSPO binding^17^^,^^64^ as compared to many more amino acids, especially in the RING domain, that are involved in the ligase activity of the ZNRF3 protein itself. Therefore, this relatively small number of critical RSPO binding sites may explain that we observed only one affected individual with microcephaly in our cohort.

Apart from the brain phenotypes, we observed some clinical findings such as nail hypoplasia, lacrimal duct obstruction, and oligodontia that were specific to the affected individual with microcephaly who harbored the RSPO binding site variant p.Leu104Pro and showed decreased Wnt signaling in the functional assay. Despite the limited number of one, this observation of phenotypes specific to decreased Wnt signaling is supported by previous studies, showing that compromised Wnt signaling can cause such phenotypes.^65^^,^^66^^,^^67^ In fact, mutations in *LEF1* (MIM: 153245), which is a transcription factor downstream of the Wnt signaling, has been linked to ectodermal dysplasia, including one individual also affected by microcephaly.^68^ Nevertheless, further observations of pathogenic variants in the RSPO binding domain are necessary to strengthen the suggested genotype-phenotype correlation.

As *ZNRF3* is considered a tumor repressor gene, further research on therapeutic interventions by Wnt modulators may be considered for affected individuals with *ZNRF3* germline mutations, especially those with macrocephaly and hence variants associated with increased Wnt signaling (Figures 2C, 3, and 4J–4T) who can have increased risk for cancer during their lifetime.^69^

In early *Xenopus* embryos, *znrf3* is highly expressed in many compartments, including Spemann organizer, midbrain, hindbrain, neural tube, branchial arches, and optic vesicle.^70^ *ZNRF3* is ubiquitously expressed during development,^61^^,^^71^^,^^72^ which reflects in anomalies observed in a variety of tissue types upon disruption of its function, such as adrenal cortex,^73^ hepatocytes,^74^ or fibroblast-like synoviocytes.^75^ Its complete knock out is not compatible with life.^5^ Apart from the many studies on *ZNRF3* in cancer tissues, several previous studies especially focused on the function of *ZNRF3* in the adrenal gland. It has been shown that in mice *Znrf3* is expressed throughout the adrenal cortex during development, and its conditional knockout resulted in adrenal hyperplasia with a reduced plasma concentration of corticosterone.^73^ This may partly support a causal link between *ZNRF3* and the adrenal insufficiency observed in the two individuals in our cohort who each harbored a large in-frame deletion in *ZNRF3* (I-10 and I-11; Table 1). This link is further supported by a recent study reporting three individuals with adrenal insufficiency,^27^ as well as an additional individual with adrenal insufficiency^76^ who harbored deletions of different sizes affecting the exon 2 of *ZNRF3*.

Taken together, our findings provide evidence for the link between *ZNRF3* and the two mirror brain phenotypes of microcephaly and macrocephaly and emphasize the role of the Wnt/β-catenin signaling in regulating proper human brain size during neurogenesis.

## Data and code availability

We have uploaded the *ZNRF3* variants reported in this study to ClinVar: SCV005050154, SCV005050155, SCV005050156, SCV005050157, SCV005050158, SCV005050159, SCV005050160, SCV005050161, SCV005050162, SCV005050163, and SCV005050164. There are restrictions to the availability of further genomic data of the individuals studied here due to the consent given by them or their legal guardians. Other data will be provided upon legitimate requests.

## Web resources

ClinVar, https://www.ncbi.nlm.nih.gov/clinvar/

COSMIC, https://cancer.sanger.ac.uk/cosmic/

DECIPHER, https://www.deciphergenomics.org/

Ensembl, https://www.ensembl.org.

GenBank, https://www.ncbi.nlm.nih.gov/genbank.

GeneCards, https://www.genecards.org.

GeneMatcher, https://genematcher.org.

Genome Aggregation Database, http://gnomad.broadinstitute.org.

Human Gene Mutation Database, https://portal.biobase-international.com/hgmd.

MetaDome web server, https://stuart.radboundumc.nl/metadome/

OMIM, http://www.omim.org.

Protein DataBank, https://www.rcsb.org.

SysNDD, https://sysndd.dbmr.unibe.ch/

UCSC Genome Browser, https://genome.ucsc.edu.

UniProt, https://www.uniprot.org.

Zenodo, https://zenodo.org/records/8208688.

## Supplemental information

Table S1. Summary of ZNRF3 variants and clinical findings

Table S2. Summary of genes in the Wnt signaling pathway

Table S3. Beta-catenin physical target genes in hESC
